# Ametropia in children with headache

**DOI:** 10.12669/pjms.35.3.268

**Published:** 2019

**Authors:** Mohammad Asim Mehboob, Haider Nisar, Memoona Khan

**Affiliations:** 1 *Dr. Mohammad Asim Mehboob, FCPS(Ophth), FICO, FRCS, MRCSEd, Combined Military Hospital, Gujranwala, Pakistan*; 2 *Dr. Haider Nisar, FCPS(Paeds), Combined Military Hospital, Khuzdar, Pakistan*; 3 *Dr. Memoona Khan, FCPS (Hematology), Combined Military Hospital, Khuzdar, Pakistan*

**Keywords:** Ametropia, Headache, Refractive error

## Abstract

**Objective:**

To measure the frequency of uncorrected ametropia in children with 2 to 8 weeks of persistent headache referred to ophthalmic outpatient department for evaluation.

**Methods:**

This cross sectional study was conducted at CMH Gujranwala from March 2018 to November 2018.A total of 262 children, aged from 5 to 16 years, with 2 to 8 weeks history of persistent headache underwent detailed ophthalmic assessment for refractive errors, and other ophthalmic evaluation. Children with ametropia, confirmed with cycloplegic refraction and post-mydriatic testing were prescribed with glasses. Patients without any ophthalmic findings were referred back to pediatrics department for further evaluation.

**Results:**

Mean age of study population was 8.97 ± 3.16 years. Mean duration of headache was 5.03 ± 1.81 weeks. Ametropia was found in 56 (21.4%) children, while 206 (78.6%) had no refractive error. Out of children with ametropia, 20 (35.7%) had myopia, 24 (42.8%) had astigmatism and 12 (21.5%) had hypermetropia. There was no difference in ametropic children and children without ametropia with respect to gender (p=0.73), age (p=0.54) and duration of headache (p=0.71).

**Conclusion:**

A significant proportion of children with ametropia have initial symptoms of headache. Any child with un-explained headache must undergo ophthalmic evaluation to diagnose refractive error, if any.

## INTRODUCTION

Patients are frequently referred to ophthalmology department from pediatric care units for evaluation of refractive error in case of headache. While there are numerous causes of headache, uncorrected or under-corrected refractive errors have long been considered a potential cause of headache in pediatric population.^1^Numerous studies have shown a significant relation between these two entities. However, the evidence base for this remains shallow, due to various studies showing no significant relationship.

Headache disorders are the most frequently encountered clinical entity. 2 Headache has a profound effect on quality of life. This significantly hampers daily routine, affects mood and cognition and often leads to severe psycho-social issues.^3^ Headache is considered a disabling condition in pediatric age group, which, if not properly managed, can lead to long term effects on child’s psychology and mental wellbeing.^4^ The international headache society has delineated headache due to refractive error in a methodical manner. It shows that headache is caused by uncorrected hyperopia or astigmatism. The headache is usually mild, more common in frontal region and is usually absent on waking up. The headache is reported to worsen with prolonged visual activity.^5^With the disease entity so specifically described, it is imperative to consider this a yardstick for evaluation of children with unexplained headache not falling into any other category. A routine ophthalmic evaluation is perhaps mandatory.

To the researcher’s knowledge, no study has been conducted in Pakistani population to measure the prevalence of uncorrected ametropia in children with persistent and un-explained headache. The aim of this study was to measure the frequency of uncorrected ametropia in children with 2 to 8 weeks of persistent headache referred to ophthalmic outpatient department for evaluation.

## METHODS

This cross sectional study was carried out at Combined Military Hospital (CMH) Gujranwala, from March 2018 to November 2018, after approval from the institutional ethical review committee, and taking written informed consents from parents of children included in the study. A total of 262 children from either gender, aged from 5 to 16 years, with 2 to 8 weeks history of persistent headache were included in the study. Children with history of squint, refractive error, head injury, trauma, intracranial space occupying lesions, sinusitis, migraine, prolonged drugs use, epilepsy, fever, and other known neurological diseases were excluded. All children had remained under evaluation of pediatrician for diagnosis of headache. Those with a certain diagnosis were also excluded from the study. All children received in ophthalmic department underwent evaluation of visual acuity, best corrected visual acuity after cycloplegic refraction, fundoscopy, assessment for squint using Hirschberg test and cover uncover test, stereopsis testing using Titmus test and detailed anterior and posterior segment examination. Those children diagnosed with ametropia were subsequently re-called for post mydriatic testing for best corrected visual acuity, and prescribed with glasses. They were re-evaluated after 4 weeks and 8 weeks of prescription of glasses for complaints of headache. Patients without any ophthalmic findings were referred back to pediatrics department for further evaluation.

The pre devised proforma was completed by researcher endorsing subject’s demography and ocular examination findings. Confidentiality of the patient’s record was maintained. Statistical Package for Social Sciences (SPSS 20.0) for windows was used for statistical analysis. Descriptive statistics i.e. mean ± standard deviation for quantitative values (age, duration of headache) and frequencies along with percentages for qualitative variables (gender, refractive errors) were used to describe the data. The children were further divided in two groups, with ametropia, and those without ametropia. After normality testing, qualitative variable like gender was compared between two groups using Chi Square test and quantitative variables like age and duration of headache were compared using independent ‘t’ test. A p-value of ≤0.005 was considered statistically significant.

## RESULTS

A total of 262 children fulfilling the inclusion criteria were evaluated. Out of 262 children, 127 (48.5%) were males, and 135 (51.5%) were female. Mean age of study population was 8.97 ± 3.16 years. Mean duration of headache was 5.03 ± 1.81 weeks. Ametropia was found in 56 (21.4%) children, while 206 (78.6%) had no refractive error. Out of children with ametropia, 20 (35.7%) had myopia, 24 (42.8%) had astigmatism and 12 (21.5%) had hypermetropia. The comparison of groups (ametropia/no ametropia) with respect to age, gender and duration of headache is given in Table-I. There was no difference in ametropic children and children without ametropia with respect to gender (p=0.73), age (p=0.54) and duration of headache (p=0.71). After correction of ametropia, 35 (62.5%) children reported alleviation of headache after 4 weeks, and 42 (75%) reported alleviation in symptoms after 8 weeks. A total of 14 (25%) children reported headache even after correction of ametropia with glasses. Further discussion about causes of headache in study population is beyond the scope of this study.

**Table-I:** Group wise demographic data (n=262).

Characteristic	Study Population (n=262)	Group-A (Ametropia) (n=56)	Group-B (No amteropia) (n=206)	p-Value
Age (Years) Mean ± SD	8.96 ± 3.16	9.19 ± 3.38	8.90 ± 3.10	0.546*
***Gender***				
Male	127 (48.5%)	26(46.4%)	101(49%)	0.730**
Female	135 (51.5%)	30(53.6%)	105(51%)	
Duration of headache (weeks) Mean ± SD	5.03 ± 1.81	4.94 ± 1.84	5.04 ± 1.80	0.709*

* Independent t-test,

** Chi Square test

## DISCUSSION

The actual reason of headache due to ametropia remains en-explained. It is believed that contraction of ciliary muscles is effortless and should not lead to headache. Study by Cameron ME revealed that very few cases with headache had refractive error. They also found that in presbyopia and hypermetropia, headaches are infrequent. They postulated that headaches observed are due to associated contraction of the scalp muscles.^6^ Medical term for headache associated with eye strain or fatigue is asthenopia, which is thought to be triggered by ametropia, extra ocular muscle imbalance, improper reading or working environment and inaccurate reading or working habits.^7^ Another meta-analysis on the subject including 2465 subjects revealed that majority of children with asthenopia had no refractive error and estimated a pooled prevalence of ametropia of 19.7% in children.^8^

Ophthalmologists are frequently referred cases for evaluation of refractive errors and diagnose ocular causes of headache. Many authors have divided causes of headache into ocular and non-ocular causes.^9^ While majority of these are related to asthenopia or refractive errors, other conditions like corneal ulcers, migraine, glaucoma, giant cell arteritis, idiopathic intracranial hypertension, sinusitis, hypertension and scleritis are also known to cause headache.^10^ This shows that all patients with headache must undergo complete ophthalmic evaluation.

The type of headache due to refractive errors varies and is mostly subjective. Some patients report a dull peri-ocular pain, while others report frontal headache of sharp nature. We included children with all sorts of headache, frontal, temporal, migraine like and cluster type. In a study by Gunes A and associates, it was found that migraine was a common type of headache in children with ametropia.^11^ Other types of headaches are also reported in relation to asthenopia. Orbell G gave hopeful results of treating children having eye strain with chlordiazepoxide in 1963.^12^ Thus headache due to eye strain is manageable, either with correction or with systemic treatment of headache.

In our study population, the prevalence of ametropia was found to be 21.4%. The total study population had included children with un-explained headache, refractory to medical treatment. In a large study including 1448 children with headache, 82.3% had a normal eye examination, while refractive errors, amblyopia, and strabismus were found in 15.0%, 3.6%, and 7.3%, respectively.^13^ In another study, headache was reported in 23.1% of schoolchildren and it was concluded that asthenopia was significantly associated with uncorrected visual acuity and myopia among schoolchildren.^14^ Another study conducted on child laborers working in gem polishing industries revealed that excessive near work lead to asthenopia or eye strain in as many as 32.2% children.^15^

We found a significant number of children with headache suffering from uncorrected ametropia. In one study, it was recommended that headaches in children usually do not appear to be caused by ophthalmic disease, including refractive error.^16^ However, other studies have clearly described that compound and mixed types of astigmatism, anisometropia, and miscorrection of refractive error were found more often in patients with headache than in control subjects.^17^Few studies comparing the children with headache with control group have concluded that headache is not associated with refractive errors, and proper correction of refractive errors significantly improved headache complaints and did so primarily by decreasing the frequency of headache episodes, rather than improvement on visual acuity.^18^,^19^ Further research in this regard will be helpful for defining a medical strategy for children with headache.

We found that out in children with ametropia, 20 (35.7%) had myopia, 24 (42.8%) had astigmatism and 12 (21.5%) had hypermetropia. This is in accordance with prevalence found by studies showing that low degrees of astigmatism and anisometropia are relevant in migraine and headache.^20^ In another study conducted in Israel, it was found that anisometropia and myopia were the most common refractive errors encountered, followed by hyperopia and astigmatism in children with headache.^21^

We also found out that after correction of ametropia, 35 (62.5%) reported alleviation of headache after 4 weeks, and 42 (75%) reported alleviation in symptoms after 8 weeks. 14 (25%) reported headache even after correction of ametropia with glasses. This is in accordance with research conducted by Jeddi A et al.^22^ However, other studies have asserted that despite the apparent belief that provision of an appropriate correction may alleviate various types of headache, there is little robust evidence in support of this position.^23^ Authors presume that alleviation in symptoms of headache is a subjective feeling, that depends on response of children to optical correction. Exact and objective assessment in this regard will always remain doubtful and will warrant large cohort trials.

The study has limitations of not including a control group, and not discussing the details of ultimate diagnosis of headache in children without ametropia. Presumably, comparison with a control group will better explain the prevalence of ametropia in children.

## CONCLUSION

We conclude that a significant majority of children with un-explained headache have refractive errors. Proper evaluation of refractive error and full optical correction can help in improvement in headache for these children. Refractive errors should always be considered in differential diagnosis of all children who present to pediatrics outpatient with un-explained and refractory headache.
